# A Four-Compartment Metabolomics Analysis of the Liver, Muscle, Serum, and Urine Response to Polytrauma with Hemorrhagic Shock following Carbohydrate Prefeed

**DOI:** 10.1371/journal.pone.0124467

**Published:** 2015-04-14

**Authors:** Nancy Witowski, Elizabeth Lusczek, Charles Determan, Daniel Lexcen, Kristine Mulier, Beverly Ostrowski, Greg Beilman

**Affiliations:** 1 Department of Surgery, University of Minnesota, Minneapolis, MN, United States of America; 2 Minnesota NMR Center, University of Minnesota, Minneapolis, MN, United States of America; Rutgers University, UNITED STATES

## Abstract

**Objective:**

Hemorrhagic shock accompanied by injury represents a major physiologic stress. Fasted animals are often used to study hemorrhagic shock (with injury). A fasted state is not guaranteed in the general human population. The objective of this study was to determine if fed animals would exhibit a different metabolic profile in response to hemorrhagic shock with trauma when compared to fasted animals.

**Methods:**

Proton (1H) NMR spectroscopy was used to determine concentrations of metabolites from four different compartments (liver, muscle, serum, urine) taken at defined time points throughout shock/injury and resuscitation. PLS-DA was performed and VIP lists established for baseline, shock and resuscitation (10 metabolites for each compartment at each time interval) on metabolomics data from surviving animals.

**Results:**

Fed status prior to the occurrence of hemorrhagic shock with injury alters the metabolic course of this trauma and potentially affects mortality. The death rate for CPF animals is higher than FS animals (47 vs 28%). The majority of deaths occur post-resuscitation suggesting reperfusion injury. The metabolomics response to shock reflects priorities evident at baseline. FS animals raise the baseline degree of proteolysis to provide additional amino acids for energy production while CPF animals rely on both glucose and, to a lesser extent, amino acids. During early resuscitation levels of metabolites associated with energy production drop, suggesting diminished demand.

**Conclusions:**

Feeding status prior to the occurrence of hemorrhagic shock with injury alters the metabolic course of this trauma and potentially affects mortality. The response to shock reflects metabolic priorities at baseline.

## Introduction

Hemorrhagic shock accompanied by injury represents a major physiologic stress. The programmed response to this stress relies on internal energy sources [1–3]. Fasted animals are often used to study hemorrhagic shock (with injury). A fasted state is not guaranteed in the general human population. Additionally the fasted state could predispose the animals to a more favorable outcome [4,5]. The objective of this study was to determine if fed animals would exhibit a different metabolic profile in response to hemorrhagic shock with trauma when compared to fasted animals. Specifically, we compared the effect of providing a carbohydrate prefeed (CPF) to an overnight fast (FS) prior to insult on the metabolome. We have previously described metabolomics changes that accompanied fed status in individual compartments [6–8]. In this work we consider data from the four compartments simultaneously produces a more complete picture. Proton (^1^H) NMR spectroscopy was used to determine concentrations of metabolites from four different compartments (liver, muscle, serum, urine) taken at defined time points throughout shock/injury and resuscitation. We hypothesized that there would be quantifiable differences in metabolites reflecting an altered response to shock and early resuscitation depending upon fed state.

## Methods

### 2.1. Animal preparation and hemorrhagic shock protocol

The experimental protocol was approved by the University of Minnesota Animal Use Committee and was conducted in accordance with established guidelines for the treatment of laboratory animals. A modification of our well-established model of porcine hemorrhagic shock was used [9,10]. Sixty-four Yorkshire pigs (Manthei Hog Farm, LLC, Elk River, MN) weighing between 15–20 kg were randomized to either a fed or fasted group. Both groups were fasted overnight but allowed free access to water for the 12 hours prior to the execution of the protocol. CPF animals were given an oral carbohydrate solution (Karo Light, 7cc/kg, diluted with equal volume of water) 60 minutes prior to the administration of initial anesthesia. [^1^H NMR of Karo Light indicated it is a mixture of mono- and di-saccharides (glucose, fructose, maltose, sucrose)].

#### 2.1.1. Instrumentation and surgical preparation

All 64 animals were anesthetized with an intramuscular dose of telazol (Wyeth Animal Health, Madison, NJ). Anesthesia was maintained by an IV infusion of propofol (2–9 mg/kg, AstraZeneca Pharmaceuticals, Wilmington, England) and 60% inhaled nitrous oxide. Upon sedation, the pigs were orally intubated and ventilated to maintain a PO_2_ of 70–120 torr and a PCO_2_ of 35–45 torr (SERVO Ventilator 900C, Siemens, Malvern, PA). Peripheral intravenous lines were placed in the surgically exposed right femoral artery and right jugular vein. A catheter was placed in the right femoral artery for continuous measurement of blood pressure and blood sampling. An introducer (7 French Avanti, Cordis Corporation, Miami Lakes, FL) was placed into the right jugular vein and a Swan-Ganz catheter (5 French, Edwards Lifesciences, Irvine, CA) was placed for measurements of pulmonary artery pressure, pulmonary wedge pressure, cardiac output, and mixed venous blood sampling. Animals then underwent a midline laparotomy and splenectomy. A Foley catheter was placed in the urinary bladder via stab cystostomy for collection of urine. The inferior vena cava (IVC) was cannulated for blood removal. After surgical preparation, animals were allowed to stabilize until plasma lactate levels reach a value of 2.0 mmol/L or less.

#### 2.1.2. Experimental trauma and shock

**Fig 1:**
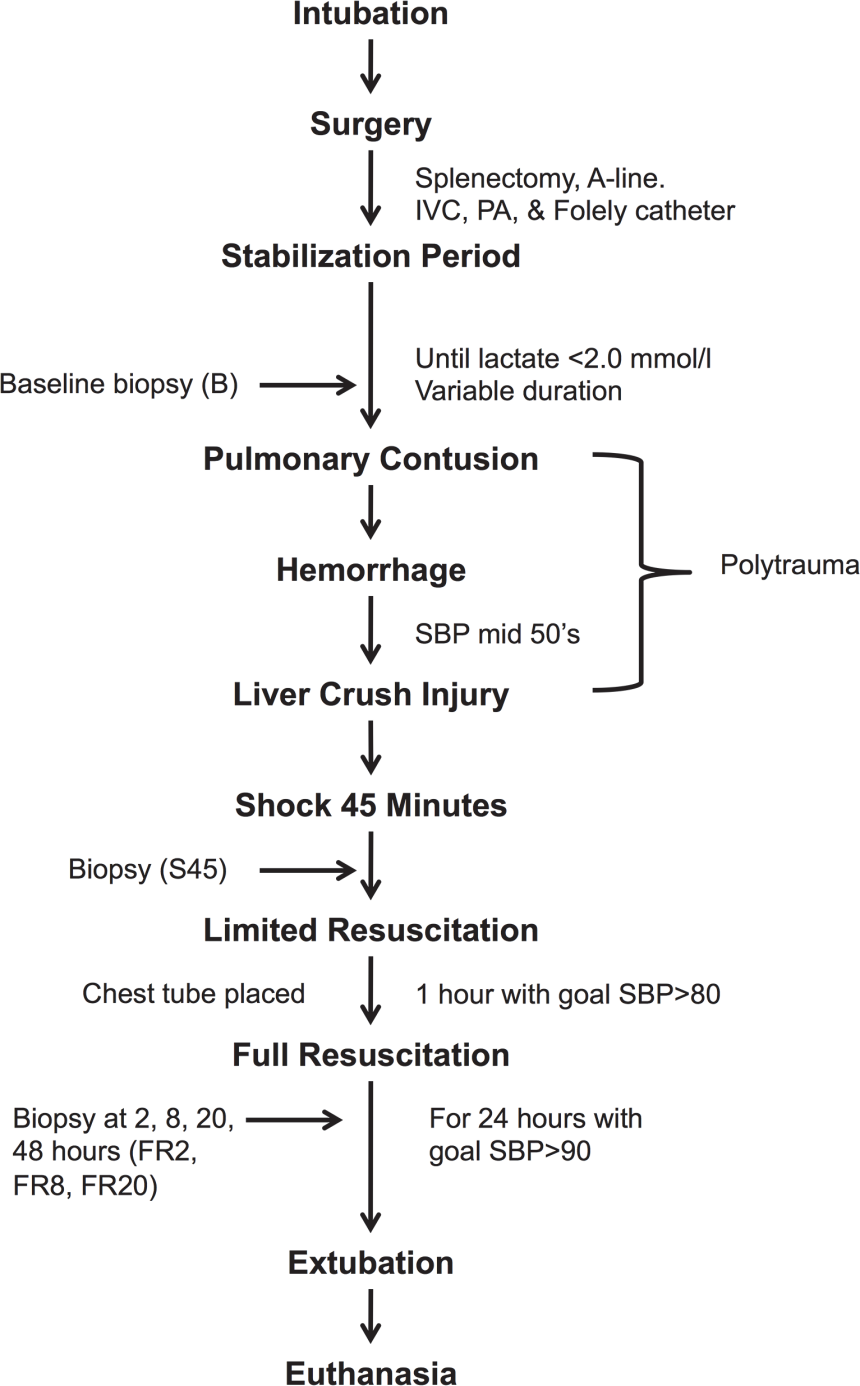
Graphical representation of experimental timeline. Experimental procedures and biopsy sampling timepoints are shown.

A captive bolt device was used to create a blunt percussive injury to the right chest. Hemorrhagic shock was then induced in these animals by withdrawal of blood from the IVC until a systolic pressure in the lower 50’s was reached (typically 35% of total blood volume). Shed blood was placed in an acid-citrate-dextrose bag for later use. A liver crush injury was induced using a Holcomb clamp technique with two crush injuries created in the liver parenchyma [11]. (Fig 1)

#### 2.1.3. Experimental resuscitation

Animals were resuscitated with lactated Ringer’s fluid given as 20 cc/kg intravenous (IV) boluses to maintain a systolic blood pressure of greater than 80 mmHg for one hour of limited resuscitation, then underwent full resuscitation by protocol. Auto-transfused warmed blood was given at 10 cc/kg IV boluses for a target hemoglobin of greater than 6 g/dL, and a urine output of greater than 1 cc/kg/hr. Lactated Ringer’s at 20 cc/kg IV boluses or blood at 10 cc/kg/hr were given as needed. After a resuscitation period of 20 hours, animals were extubated and sent for recovery in which they received a standard diet. At 48 hours after resuscitation, animals were re-intubated for endpoint sample harvesting and then euthanized with Beuthanasia D (1 ml/10kg IV).

### 2.2. Biological sampling

Numerous physiologic parameters as well as serum, urine, and tissue (muscle and liver) samples were collected at multiple time points including baseline (B), after 45 minutes of shock (S45), 2 (FR2), 8 (FR8), and 20 (FR20) hours after the initiation of full resuscitation, and 48 hours after the beginning of the shock phase (PR48). (Fig 1) Blood glucose, lactate, and blood urea nitrogen were measured using a Gem Premier 3000 blood gas analyzer (Instrumentation Laboratories, Chicago, IL). Additional laboratory testing analyzed collected plasma samples for (among others): alanine aminotransferase, aspartate aminotransferase, total bilirubin, lactate dehydrogenase, and alkaline phosphatase. Liver, muscle, serum and urine samples were stored at -80°C until NMR analysis.

### 2.3. NMR

#### 2.3.1. Liver and muscle water-soluble metabolite preparation

Muscle and liver samples were processed for NMR analysis according to established protocol [12]. Frozen biopsy specimens were pulverized to a fine powder using a liquid nitrogen-chilled mortar and pestle. Ice-cold perchloric acid (6%, 5 mL/g wet weight) was added to the pulverized tissue (100–150 mg wet wt), and the sample vortexed for 30 seconds. Following a10 minute incubation on ice, samples were centrifuged (14,000 rpm, 4°C), the pH of the supernatant adjusted to between 7 and 8 with 2M K_2_CO_3_ and the neutralized samples incubated on ice for 30 min. The KClO4 precipitate was removed by centrifugation. The resulting supernatant was lyophilized (LABCONCO FreeZone bulk tray dryer (Kansas City, MO) and stored at ___80°C until NMR analysis.

#### 2.3.2. NMR spectroscopy of tissue samples

Lyophilized samples were reconstituted in 500 μl D_2_O (D_2_O, Aldrich, 99.9%); 50 μl of 3 mM 2,2-dimethyl-2- silapentane-5-sulfonate (DSS, Cambridge Labs) were added to serve as a lock signal and chemical shift references (δ = 0.0). Solution pH was adjusted with DCl and NaOD to 7.4. Final volume was brought to 600 μL with D_2_O making the solution 25 mM in DSS and the sample was transferred to a 5mm NMR tube (Wilmad, Vineland, NJ).

^1^H NMR data acquisition was performed at 600 MHz using a Varian spectrometer with a 5mm HCN triple resonance probe at 25°C. Muscle spectra were generated from 128 scans with a basic ^1^H acquisition protocol consisting of a 45° tip angle, a relaxation delay of 12.8 sec and an acquisition time of 7 sec. Liver spectra were generated from 1028 scans with a basic ^1^H acquisition protocol consisting of a 45° tip angle, a relaxation delay of 2 sec and an acquisition time of 1.9 sec. Phase and baselines were corrected manually before integration. Chemical shifts were assigned relative to the internal standard signal at 0 ppm.

#### 2.3.3. Sample preparation and NMR spectroscopy of serum samples

Five hundred microliters of thawed serum were mixed with 50 μl of 1 mM trimethylsilylpropionic acid (TSP, Sigma-Aldrich). (TSP served as a lock signal and chemical shift references (δ = 0.0)]. Sample pH measured ~8 and was not further adjusted. The sample was transferred to a 5mm NMR tube [13].

One-dimensional 1H-NMR spectra were acquired on a 700-MHz Bruker Avance NMR spectrometer with a 5-mm TXI proton-enhanced cryoprobe. A Carr-Purcell-Meiboom-Gill presaturation (CPMG) pulse sequence with a spectral width of 10 kHz was used to acquire all spectra with 128 scans, which were subsequently phase and baseline corrected [13]. Chemical shifts were assigned relative to the internal standard signal at 0 ppm.

#### 2.3.4. NMR Spectroscopy of urine samples

One mL of thawed urine was mixed with 0.5 mL of 0.2 M sodium phosphate buffer. The solution was placed on ice for 10 min and then centrifuged at 7000×g for 10 min. 500 μL of the supernatant was withdrawn and combined with 50 μL of 1mM TSP [14]. [TSP served as a lock signal and chemical shift references (δ = 0.0).] The pH of the final solution was recorded and the mixture was transferred to a 5 mm NMR tube.

Proton NMR spectra were taken with a Bruker Avance spectrometer with autosampler and 5 mm triple resonance 1H/13C/15 N TXI CryoProbe with Z-gradient, running TopSpin v. 2.16 (Bruker BioSpin, Fremont, CA USA) at 700.13 MHz. A 1D NOESY (Nuclear Overhauser Effect Spectroscopy) pulse sequence was used to collect spectra of each sample. The 90° pulse width was calibrated for each sample, and was generally 12–13 μs. The relaxation time was defined by each sample’s 90° pulse width. The relaxation delay was 2 s, the acquisition time was 3 s, the spectral width was 10 kHz, the total number of data points collected was 63,000, and the number of transients collected was 128, for a total experiment time of 11 min and 17 s. During the relaxation period, the water resonance was presaturated. All spectra were collected at a temperature of 298 K. Line broadening at 0.5 Hz was applied before FFT; autophasing and auto-baseline correction were applied by TopSpin [13].

#### 2.3.5. Spectral profiling and quantification

Spectra from each compartment were fit using Chenomx NMR Suite version 7.0 (Edmonton, Alberta, Canada) [15]. Fine manual phasing and baseline corrections were applied to each spectrum before targeted profiling was performed. The identification and assignment of all metabolites was based on chemical shift relative to the designated internal standard and comparison with the published literature including the spectral library available from Chenomx and in the Human Metabolome Database (www.hmdb.ca). Tissue metabolites (51 in the liver; 39 in the muscle) are reported as mM/g lyophilized tissue. Serum metabolites (53) are reported in mM. For the urine (60) metabolite concentrations were multiplied by urine output (cc/hour/kg) to correct for changes in the concentration of urine throughout the experiment [16]. The final urine metabolite abundances (nmol/h/kg) were log-transformed (base 10) to allow for comparisons among metabolites over several orders of magnitude (range: 0–5.9 × 10^5^ nmol/h/kg). To address taking the logarithm of zero, 0.1 was added to the normalized data. The range of the log-transformed, normalized urine data was [−1, 5.77]. Urea was removed from the urine and serum data sets because its signal was compromised by the water suppression used in the CMPG and NOESY pulse sequences.

### 2.4. Statistical analysis

A preliminary analysis of all the data indicated that the feeding vs fasting results were confounded with mortality. Thus, we opted in this manuscript to restrict the analysis to those animals that survived within each group to the end of the experiment.

A multivariate approach was used to analyze each of the time points. To determine the response to shock, differences were analyzed between each subsequent time point in addition to comparing Baseline levels. All statistical analysis was conducted with the open source R statistical program [17]. For each time point/difference the profiled metabolites were auto-scaled and mean-centered prior to initial principal component analysis (PCA). Samples that fell outside a 95% Hotelling’s ellipse of a scores plot of the first two principal components were considered outliers and removed from further analysis. Datasets with outliers removed were subsequently analyzed by partial least squares discriminant analysis (PLS-DA), a common discrimination technique utilized in metabolomics [18–20] that has been successfully implemented previously in our lab [7,8]. The R packages DiscriMiner [21] and permute [22] were used collectively to conduct the PLS-DA model, cross-validation, and permutation tests. PLS-DA models were optimized on the number of misclassifications (NMC) that has been show to be more powerful than other indicators, such as Q^2^, at detecting differences between groups [23]. Cross-validation was conducted via cross-model validation, i.e. nested-CV, 2CV [24], wherein the dataset is randomly split into training and testing datasets (75%, 25%). PLS-DA models were generated from the training dataset and a leave-10-out internal cross-validation. Optimized models were then used to predict the testing set (outer cross-validation). Prediction accuracy was calculated from the resulting confusion matrix. Models with accuracy ≥ 80% were considered potential models. Model quality assessment was assessed by random permutation of group class with 1000 iterations where a low permutation p-value (p ≤ 0.05) indicated a strong model. Metabolites were subsequently ranked according to their respective variable importance of projection (VIP) score. Metabolites that achieved a VIP score of 1.0 or higher, with a maximum of 10, were regarded as the primary drivers of the calculated discrimination. A student’s t-test assuming unequal variances was used to evaluate the significance level of differences in serum urea (laboratory test). Survival analysis was conducted using the logrank test on all animals.

## Results

### 3.1. Physiology

The death rate for CPF animals was higher than FS animals (47 vs 28%, p = 0.153) [25].

### 3.2. Metabolomics model analysis

A table of all profiled metabolites is provided in the supplemental materials (S1 Table). Diagnostics for PLS-DA models created to discriminate by group (CPF vs FS) for each of the four compartments at early time periods (B, S45-B, FR2-S45) are reported in Table 1. According to the criteria stated above, liver, serum, and urine at baseline are potentially discriminatory. During the S45-B time interval, liver and serum are potentially discriminatory. During the FR2-S45 time interval, liver, muscle and serum are potentially discriminatory. The models with the greatest predictive power between groups (p≤ 0.05) are urine at baseline, liver at FR2-S45, and serum at FR2-S45. Serum at baseline and liver at S45-B are nearly significant models, with p = 0.06. Model diagnostics for later time intervals are reported in S2 Table.

**Table 1:** PLS-DA model diagnostics.

Model	Compartment	R^2^	NMC ± std. dev.	Classification Accuracy	Permutation p-value
Baseline	Liver	0.728	1.747±1.04	97.22%	0.028
	Muscle	0.497	3.079±1.25	86.84%	0.864
	Serum	0.571	2.640±1.24	94.87%	0.148
	Urine	0.731	1.146±0.97	100%	0.001
S45-B	Liver	0.641	2.481±1.20	97.22%	0.035
	Muscle	0.443	3.209±1.25	84.21%	0.959
	Serum	0.658	2.208±1.16	100%	0.003
	Urine	0.448	2.517±1.15	94.29%	0.145
FR2-S45	Liver	0.775	2.049±1.14	97.22%	0.027
	Muscle	0.624	2.143±1.25	91.89%	0.411
	Serum	0.687	2.001±1.04	100%	0.004
	Urine	0.427	2.980±1.22	86.11%	0.873

Diagnostics for PLS-DA models constructed for each physiological compartment and each timepoint or time interval discussed. Diagnostics reported are: R^2^ (indicative of the predictive utility of the model), NMC (number of misclassifications, indicative of the number of samples misclassified as FS or CPF and standard deviation), classification accuracy (indicative of the overall accuracy of sample classification as FS or CPF), and permutation p-value (indicative of model significance). Diagnostics for the time intervals FR8-FR2 and FR20-FR8 are reported in S1 Table.

**Fig 2:**
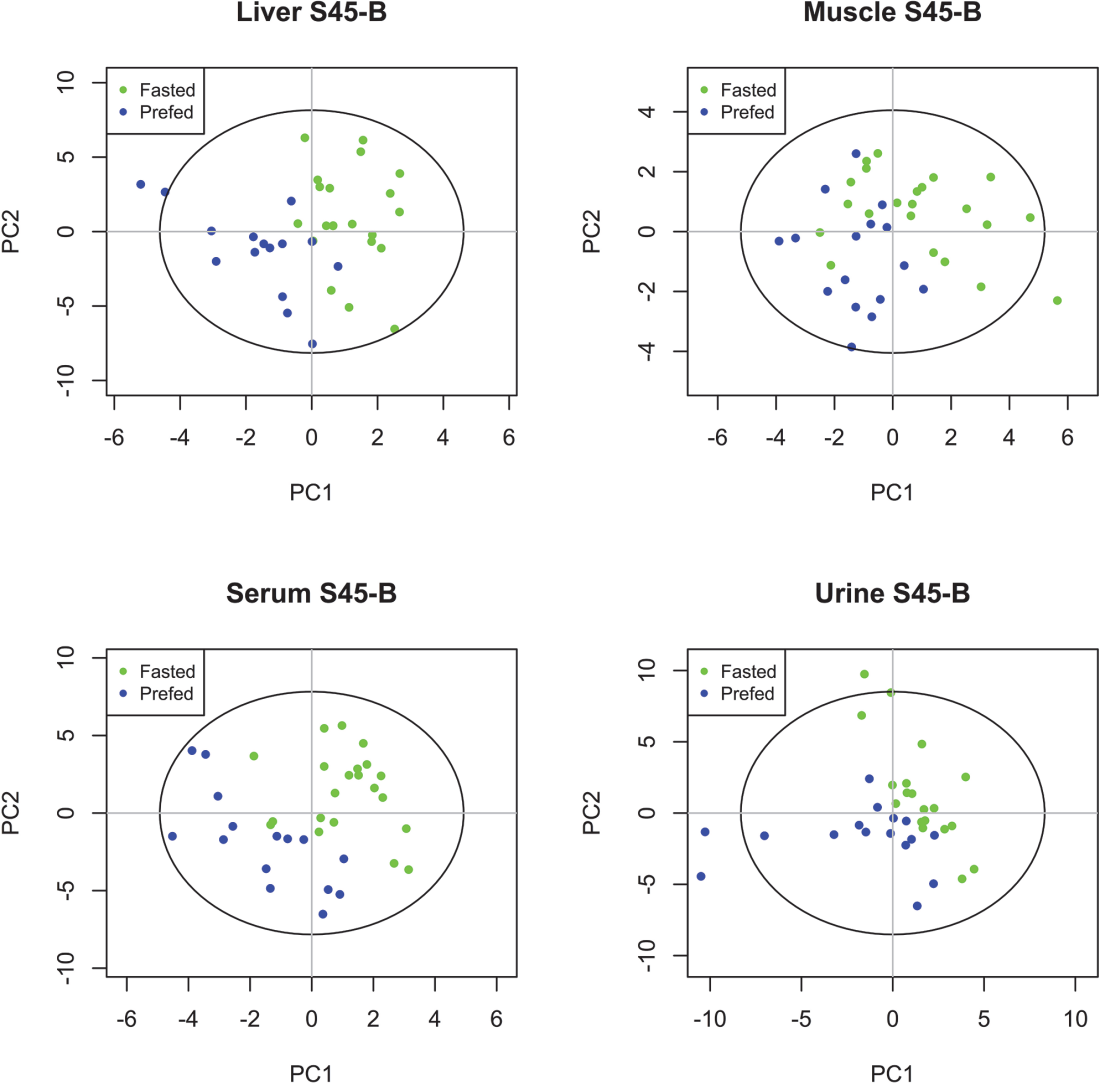
PLS-DA scores plots for the S45-B time interval. PLS-DA scores plots show model discrimination between FS and CPF animals during the response to shock (S45-B) in each of the four compartments (liver, muscle, serum, urine). Models are of varying quality and statistical significance as reported in Table 1 but indicate that there is a difference in response to shock according to feeding status.

**Fig 3:**
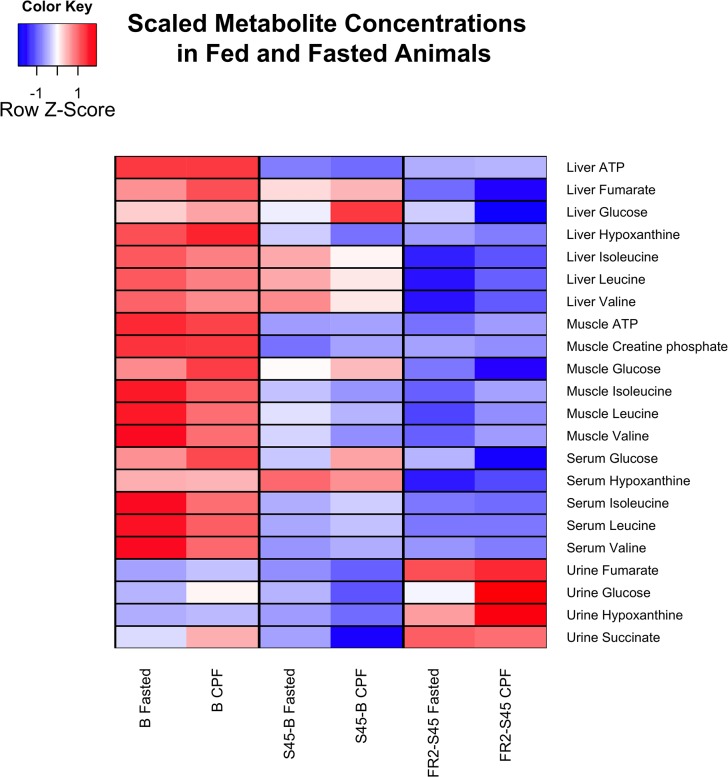
Heatmap of key differences in energy dependence between fasted and fed animals. Metabolomics data from four compartments demonstrates that animals enter the experiment in different metabolic states depending on feeding status (FS or CPF). At baseline (leftmost column), FS animals show a reliance on internal reserves demonstrated by liver, muscle, and serum levels of BCAAs while CPF animals process glucose from the pre-feed. During the response to shock (middle column), both groups demonstrate an increased reliance on internal fuel reserves. Increases in glucose in liver, muscle, and serum show mobilization of glucose in both groups for use as fuel. Evidence of increased proteolysis is demonstrated as well in increasing levels of BCAAs. During resuscitation (rightmost column), the reliance on fuel resources is diminished in both groups, with greater ATP degradation in CPF animals.

Scores plots of PLS-DA models discriminating FS from CPF for each compartment during the response to shock (S45-B) are shown in Fig 2. The scores plots for baseline and the remaining time intervals are reported in S1, S2, S3 and S4 Figs. VIP metabolites for each compartment at baseline and during the response to shock and response to early resuscitation are reported in Table 2. A heatmap of scaled concentrations of key VIP metabolites for these timepoints is shown in Fig 3. A list of VIP metabolites for later time intervals are reported in S3 Table.

**Table 2:** VIP (variable importance in projection) metabolites.

**Table 2A**	**Baseline**		**S45-B**		**FR2-S45**	
**Liver VIP Metabolite**	**CPF**	**FS**	**CPF**	**FS**	**CPF**	**FS**
maltose	**3.174**	**0.587**	**-1.245**	**-0.329**	-0.315	0.105
sucrose	**1.791**	**0.400**	**-0.709**	**-0.243**	-0.218	0.055
glucose	**7.667**	**5.241**	**15.402**	**1.136**	**-16.094**	**-1.015**
NADP^+^	**0.200**	**0.149**	**-0.045**	**-0.006**	0.013	0.023
glutamine	**1.629**	**1.071**	-0.356	-0.160	0.293	0.383
valine	**0.218**	**0.272**	**0.108**	**0.222**	**-0.117**	**-0.200**
O-phosphocholine	**2.379**	**1.758**	-0.110	-0.217	0.0980	0.227
leucine	**0.253**	**0.311**	0.111	0.193	**-0.129**	**-0.239**
NAD	**0.560**	**0.486**	0.005	0.060	-0.033	-0.082
isoleucine	**0.139**	**0.169**	**0.057**	**0.110**	-0.068	-0.104
lactate	1.832	1.479	**3.293**	**1.761**	**-2.373**	**-0.777**
asparagine	0.398	0.341	**0.138**	**-0.062**	0.011	0.039
hypoxanthine	0.213	0.181	**-0.016**	**0.042**	-0.007	0.011
dimethylamine	0.061	0.047	**0.022**	**0.000**	-0.019	-0.002
benzoate	0.194	0.200	-0.016	-0.056	**-0.029**	**0.071**
3-hydroxyisovalerate	0.068	0.077	0.003	-0.003	**-0.003**	**0.014**
fumarate	0.050	0.037	0.030	0.024	**-0.027**	**-0.008**
adenosine	0.176	0.192	0.064	0.027	**-0.058**	**0.003**
taurine	1.199	1.058	0.501	0.199	**-0.620**	**-0.220**
alanine	1.691	1.856	2.037	2.081	**-0.539**	**-1.556**
**Table 2B**	**Baseline**		**S45-B**		**FR2-S45**	
**Muscle VIP Metabolite**	**CPF**	**FS**	**CPF**	**FS**	**CPF**	**FS**
valine	**0.149**	**0.202**	**0.025**	**0.058**	**0.029**	**-0.002**
leucine	**0.114**	**0.153**	0.030	0.047	**0.016**	**-0.016**
isoleucine	**0.095**	**0.120**	**0.013**	**0.027**	**0.017**	**-0.004**
glucose	**2.757**	**1.947**	**1.468**	**0.889**	**-1.387**	**-0.360**
3-hydroxybutyrate	**0.022**	**0.038**	0.008	-0.001	0.022	0.037
imidazole	**0.145**	**0.179**	0.132	0.198	**-0.030**	**-0.184**
succinate	**0.068**	**0.084**	0.020	0.029	0.006	-0.032
ATP	**4.091**	**4.588**	0.031	-0.038	**-0.026**	**-0.702**
formate	0.191	0.218	**-0.015**	**0.081**	0.042	-0.033
creatine phosphate	16.541	16.926	**-0.927**	**-3.850**	-1.904	-0.789
fumarate	0.023	0.028	**0.008**	**0.015**	**0.005**	**-0.020**
alanine	2.458	2.329	**0.537**	**0.942**	**0.487**	**0.035**
pantothenate	0.089	0.084	**0.013**	**0.026**	0.004	-0.008
acetate	0.290	0.461	**0.005**	**0.117**	0.013	-0.015
α-ketoglutarate	0.476	0.485	**0.089**	**-0.032**	0.017	-0.014
myo-Inositol	0.826	0.841	0.109	0.203	**0.161**	**-0.066**
creatine	15.258	16.900	-0.638	0.914	**0.010**	**-2.954**
**Table 2C**	**Baseline**		**S45-B**		**FR2-S45**	
**Serum Metabolite**	**CPF**	**FS**	**CPF**	**FS**	**CPF**	**FS**
3-methyl-2-oxovalerate	**0.023**	**0.032**	**0.007**	**0.0002**	-0.0047	-0.0052
acetate	**0.183**	**0.300**	**0.168**	**0.235**	-0.099	-0.158
glucose	**39.988**	**27.059**	**23.318**	**-0.811**	**-36.968**	**-3.355**
isobutyrate	**0.020**	**0.030**	0.008	0.014	**0.003**	**0.014**
isoleucine	**0.353**	**0.515**	0.064	0.015	-0.082	-0.063
leucine	**0.730**	**0.998**	0.055	-0.023	-0.166	-0.170
serine	**0.580**	**0.756**	0.114	0.015	-0.040	-0.142
threonine	**0.453**	**0.721**	0.040	-0.038	0.006	0.014
tyrosine	**0.180**	**0.257**	0.058	0.038	-0.007	0.018
valine	**0.783**	**1.115**	0.052	-0.037	-0.114	-0.039
2-oxovalerate	0.287	0.334	**-0.005**	**-0.103**	-0.081	-0.033
3-hydroxyisovalerate	0.053	0.067	**0.005**	**-0.012**	-0.010	-0.002
acetoacetate	0.072	0.103	**-0.002**	**-0.022**	**-0.002**	**0.207**
choline	0.136	0.144	**0.063**	**0.130**	-0.065	-0.113
adipate	0.340	0.403	**-0.039**	**-0.162**	-0.060	-0.004
histidine	0.221	0.298	**-0.0162**	**-0.047**	0.027	0.036
proline	0.850	0.949	**-0.008**	**-0.149**	**0.144**	**0.458**
2-hydroxybutyrate	0.352	0.486	-0.037	0.003	**-0.007**	**-0.106**
citrate	0.130	0.146	0.052	0.014	**0.011**	**0.154**
creatine	0.621	0.712	0.296	0.307	**-0.063**	**0.322**
dimethylamine	0.037	0.045	0.001	-0.005	**-0.006**	**0.001**
lactate	6.247	6.975	12.958	12.282	**-10.386**	**-4.643**
pyruvate	0.427	0.459	0.253	0.223	**-0.099**	**0.115**
urea (lab value)*	**5.882**	**7.478**	1.563	2.304	1.06	1.27
**Table 2D**	**Baseline**		**S45-B**		**FR2-S45**	
**Urine Metabolite**	**CPF**	**FS**	**CPF**	**FS**	**CPF**	**FS**
1,6-anhydro-β-D-glucose	**1970**	**59**	-67	-16	**921**	**21**
α-ketoglutarate	**439**	**134**	**-413**	**-64**	734	755
betaine	**186**	**57**	**-110**	**5**	395	307
choline	**188**	**82**	6	24	**895**	**1783**
fumarate	**58**	**15**	**-40**	**-6**	225	195
glucose	**23,231**	**474**	**-14,492**	**-127**	**57,165**	**9648**
lactate	**394**	**76**	-571	-18	23,745	31,318
mannose	**126**	**50**	**-89**	**-23**	**375**	**100**
pyruvate	**48**	**32**	**-37**	**-5**	847	900
succinate	**188**	**43**	**-134**	**-18**	141	156
citrate	1229	816	**-943**	**-327**	2130	2153
creatine	250	135	**-137**	**33**	292	326
hypoxanthine	120	68	**-108**	**-9**	**836**	**418**
acetate	56	40	-29	-15	**105**	**209**
creatinine	3369	3842	-1401	-975	**1218**	**54**
lactose	211	240	-52	-43	**312**	**140**
mannitol	887	1172	-424	-351	**371**	**-85**
trimethylamine-N-oxide	289	179	-122	4	**328**	**94**

Mean liver (2A) and muscle (2B) values are reported as mM/1 g lyophilyzed muscle tissue. Mean serum (2C) values are reported as mmol/L. The lab values of urea (obtained with a Gem Premier 3000 blood gas analyzer) are reported instead of NMR values since the water suppression in the CPMG pulse sequence compromises the urea signal. Mean urine (2D) values are reported as nmol/hr/kg. Bolded numbers indicate metabolites that achieved VIP scores above 1.0 or were the top 10 metabolites of those metabolites with VIP scores above 1.0. Only the Baseline serum urea (laboratory analysis) reached statistical significance (p = 0.003, Student T test assuming unequal variance).

### 3.2.1. Baseline VIP metabolites.

Liver (permutation p = 0.005)

Branched chain amino acid (BCAA, leucine, isoleucine, valine) levels are higher and glucose levels are lower in FS animals compared to CPF animals. Levels of NADP^+^, NAD^+^, O-phosphocholine and the amino acid glutamine are higher in CPF animals. Constituent sugars of the Karo syrup pre-feed (maltose, sucrose) are substantially higher in CPF animals when compared to FS animals. ATP levels are not differentiating.

Muscle (permutation p = 0.869)

Branched chain amino acids, 3-hydroxybutyrate, succinate, and imidazole levels are higher in FS animals when compared to CPF animals. Levels of glucose are lower in FS animals compared to CPF animals. ATP levels are differentiating in the muscle and are lower in CPF animals when compared to FS animals. Only eight metabolites reached VIP significance of 1.00.

Serum (permutation p = 0.157)

Nine of the ten VIP metabolites are greater in the serum of FS animals when compared to CPF animals: isoleucine, leucine, valine, 3-methyl-2-oxovalerate, acetate, isobutyrate, serine, threonine, and tyrosine. FS animals exhibit higher levels of urea when compared to CPF animals. Glucose is the only metabolite to exhibit higher levels in the serum of CPF animals when compared to FS animals at baseline.

Urine (permutation p = 0.005)

The difference between experimental groups at baseline is highlighted by the high CPF levels of glucose and glucose-related metabolites (mannose, 1,6-anhydro-β-D-glucose, and the glycolytic end product, lactate). Levels of α-ketoglutarate, fumarate, pyruvate, and succinate are all higher in CPF animals compared to FS animals. Betaine and choline levels are also greater in CPF animals.

### 3.2.2. Response to shock (S45-B) VIP metabolites.

Liver (permutation p = 0.005)

Levels of BCAA rise in both groups with the increase being greater in FS animals when compared to CPF animals. (Leucine VIP = 1.08 but not in the top 10.) Levels of glucose and lactate also exhibit increases over baseline with the increases in CPF glucose and lactate being 15 times and ~2 times (respectively) that observed in FS animals. Levels of maltose and sucrose decline in both groups when compared to baseline (CPF decrease > FS decrease). Levels of NADP^+^, asparagine, hypoxanthine, and dimethylamine all change when compared to baseline with the magnitude and direction of change varying for each group comparison. ATP levels decrease in both groups with the larger decrease observed in the CPF group when compared to the FS group (-0.061 vs -0.038 mM/g lyophilized tissue, respectively). ATP levels are not differentiating and therefore not listed in the top 10 VIP metabolites.

Muscle (permutation p = 0.964)

Both CPF and FS animals exhibit an increase in levels of valine, leucine, and isoleucine (FS > CPF) when compared to baseline. (Isoleucine VIP = 1.06 but not in the top 10.) Glucose levels in CPF animals increase to a greater extent than in FS animals. Differing degrees of change from baseline are observed when FS animals are compared to CPF animals in the following metabolites: formate, creatine phosphate, fumarate, alanine, pantothenate, acetate, and α-ketoglutarate. While levels of ATP increase in CPF animals and decrease in FS animals when compared to baseline, the change in levels is not differentiating.

Serum (permutation p = 0.005)

Glucose levels increase in CPF animals and decrease in FS animals over baseline. Levels of 3-methyl-2-oxovalerate, acetate, 2-oxovalarte, 3-hydroxyvalerate, acetoacetate, choline, adipate, histidine, and proline exhibit changes when compared to baseline with the magnitude and direction of change varying for each group comparison.

Urine (permutation p = 0.107)

During episodes of blood loss, physiological mechanisms are activated to retain fluid. Although urine volumes are normalized (See Methods), interpretation of data during this time interval as well as the subsequent one (FR2-S45) should be made with this aspect in mind.

Levels of eight of the ten VIP metabolites (glucose, mannose, pyruvate, citrate, α-ketoglutarate, succinate, fumarate, and hypoxanthine) exhibit a decrease from baseline in both groups; the decrease is larger in every case in the CPF group. Levels of betaine and creatine also decrease in the CPF animals but increase in the FS animals when compared to baseline.

### 3.2.3. Response to resuscitation (FR2-S45) VIP metabolites.

Liver (permutation p = 0.042)

Both CPF and FS animals exhibit a decrease in levels of BCAA (FS decrease > CPF decrease) when compared to S45. (Isoleucine VIP = 0.99.) Glucose and lactate levels in CPF animals decrease to a greater extent than in FS animals. Differing degrees of change from S45 are observed when FS animals are compared to CPF animals in the following metabolites: benzoate, 3-hydroxyvalerate, fumarate, adenosine, taurine, and alanine. ATP levels increase to a small extent in both groups (CPF ATP = +0.017; FS ATP = +0.012 mM/g lyophilized tissue, respectively), but the levels are not differentiating.

Muscle (permutation p = 0.437)

Levels of BCAA increase in CPF animals while they decrease in FS animals compared to S45. Levels of glucose decrease in both groups (CPF decrease > FS decrease). Creatine levels increase in CPF animals while decreasing in FS animals. Imidazole, fumarate, alanine, and myo-inositol display changes in FR2 levels when compared to S45 levels and the degree and direction of change varies for each metabolite. ATP levels are differentiating in the muscle during the response to resuscitation; both groups of animals exhibit a decrease in levels but the decrease is approximately 25 times greater in FS animals when compared to CPF animals.

Serum (permutation p = 0.005)

As seen in the tissues, serum glucose and lactate levels decrease in both groups compared to S45 (CPF decrease > FS decrease). Levels of 2-hydroxybutyrate also decrease compared to S45 but the decrease is greater in FS animals compared to CPF animals. Levels of isobutyrate, proline, and citrate increase in both groups (FS increase > CPF increase). FS levels of acetoacetate, creatine, dimethylamine, and pyruvate increase while CPF levels decrease.

Urine (model significance: p = 0.857)

Levels of 1,6-anhydro-β-D-glucose, glucose, mannose, hypoxanthine, creatinine, lactose, and trimethylamine-N-oxide increase in both groups compared to S45 with the increases being greater in CPF animals. Levels of mannitol also increase in CPF animals compared to S45 while levels decrease in FS animals compared to S45. Levels of acetate and choline increase over S45 in FS animals to a greater extent than the increase observed in CPF animals.

## Discussion

In this set of experiments, we used a porcine model of polytrauma and hemorrhagic shock to investigate the metabolomics associated with pre-trauma carbohydrate feeding vs. overnight fasting in surviving animals. In this paper we report the metabolic differences observed from baseline through early resuscitation. [Due to the scope of the data set, a discussion of later resuscitation time points (FR2 through FR20) will be disseminated at a future time.] PLS-DA of the metabolic profiles from four physiologic compartments (liver, muscle, serum, urine) demonstrates differing responses to trauma and early resuscitation according to feeding group. This discussion interprets and, where possible, integrates the results from each of the four compartments. We recognize that the complexity of biological systems renders any interpretation open to debate. Nonetheless, we have used widely accepted tenets of ischemia and metabolism to guide the interpretation of our data.

### 4.1. Baseline

**Fig 4:**
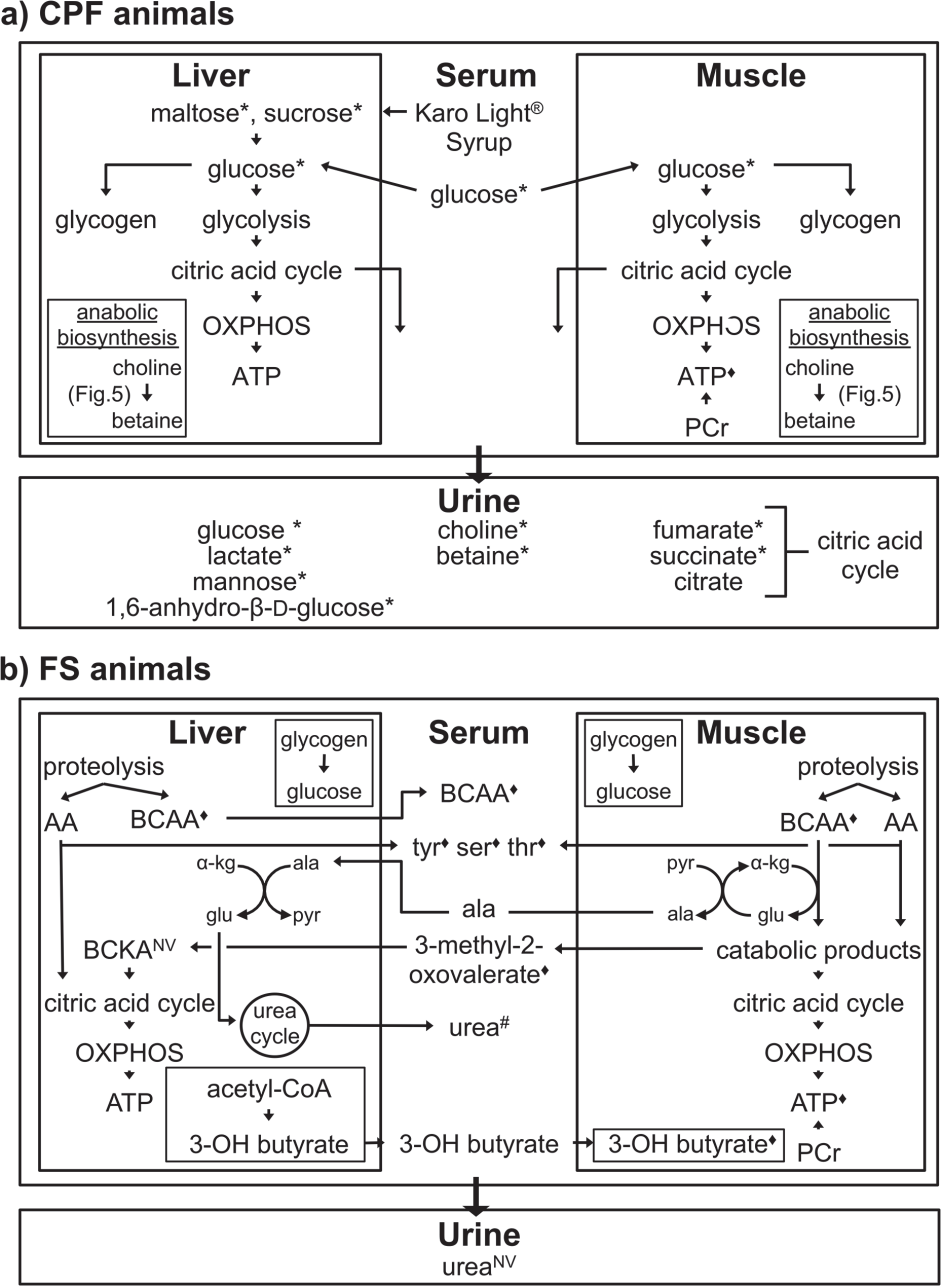
Metabolic profile associated with feeding status at Baseline. a) Higher levels of glucose are observed in liver, muscle, and serum of CPF animals. Urine choline and betaine as well as a reduced level of muscle ATP support the hypothesis of enhanced glucose-associated biosynthesis. Formation of a mitochondrial megachannel that allows for exit of citric acid cycle intermediates is suggested by elevated levels of fumarate, succinate, and citrate in the urine of CPF animals. b) Levels of liver, muscle, and serum BCAAs (isoleucine, leucine, and valine) as well as serum AA (tyrosine, serine, and threonine) are all higher in fasted animals at baseline suggesting increased proteolysis. The difference in the levels of serum urea (#) approach significance (p = 0.09) further supporting this hypothesis. * = VIP metabolite where levels observed in CPF animals > than that observed in FS animals. ◆ = VIP metabolite where levels observed in FS animals > than that observed in CPF animals. NV = metabolite not visible in that compartment.

The major findings reveal differing energy dependencies with surviving fasted animals relying on internal fuel reserves such as branched chain amino acids while surviving pre-fed animals process the external carbohydrate source (Fig 4). The presence of citric acid cycle intermediates in CPF urine implies that the capacity of the electron transport chain was exceeded as a result of the pre-feed. Glucose availability appears to promote anabolic activity in the liver.

Baseline values of VIP metabolites reflect the fed status of each group. Higher levels of maltose and sucrose in the liver of CPF animals arise from the metabolism of the carbohydrate pre-feed (Karo syrup). As would be anticipated from a pre-surgical carbohydrate feed, glucose levels are higher in all four compartments at baseline in pre-fed animals. The presence of glucose and lactate as well as the non-glycolytic end products of glucose (mannose and 1,6-anhydro-β-D-glucose) in the urine suggest that the ingested carbohydrate load was greater that the physiologic capacity to aerobically process the load.

Under high glucose-load conditions, sustained activity of the citric acid cycle results in maximal ATP levels. The increased ATP/ADP ratio inhibits F_1_F_0_ ATP synthase and produces a high mitochondrial membrane potential. As a result, electron transport is temporarily inhibited [26]. This scenario can result in the accumulation and release of citric acid cycle intermediates from the mitochondria with eventual transport into the blood for use by other tissues or for excretion [26–28]. To the best of our knowledge, the detailed mechanism by which transport out of the mitochondria is achieved has not been described. A potential explanation for this process is the formation of a megachannel in the mitochondria. This megachannel, also known as the mitochondrial permeability pore, forms in the mitochondrial membrane and allows for passage of low molecular weight moieties out of as well as into the mitochondria [29–31]. Baseline levels of several citric acid cycle intermediates (pyruvate, citrate, α-ketoglutarate, succinate, and fumarate) are higher by up to 3-fold in the urine of CPF animals compared to FS animals. This suggests that in CPF animals the capacity of the electron transport chain was exceeded and a megachannel opened allowing for these intermediates to leak out of cells. The time differential between the pre-feed and baseline sample collection may account for non-differentiating citric acid cycle intermediates in the other three compartments.

Conversely, overnight fasting initiates a shift to internal fuel sources, specifically amino acids derived from proteolysis. Degradation of amino acids produces end products that enter the citric acid cycle for the eventual production of ATP. Levels of leucine, isoleucine, and valine (branched chain amino acids, BCAA) are higher in the liver, muscle, and serum of fasted animals when compared to CPF animals. (BCAAs were not observed in the urine.) Serum levels of the amino acids tyrosine, threonine, and serine as well as 3-methyl-2-oxovalerate (the ketoacid derived from isoleucine) are also greater in FS animals when compared to CPF animals. Higher levels of urea are also observed in the serum of FS animals. Urea is produced through a series of reactions that are initiated with the transfer of a liberated amino group to glutamate. Rather than being an intermediate in the production of urea, glutamate can be enzymatically converted to glutamine. In the liver of CPF animals levels of glutamine are greater when compared to FS animals. These lines of evidence strongly support the use of amino acids as fuel in the fasted state.

**Fig 5:**
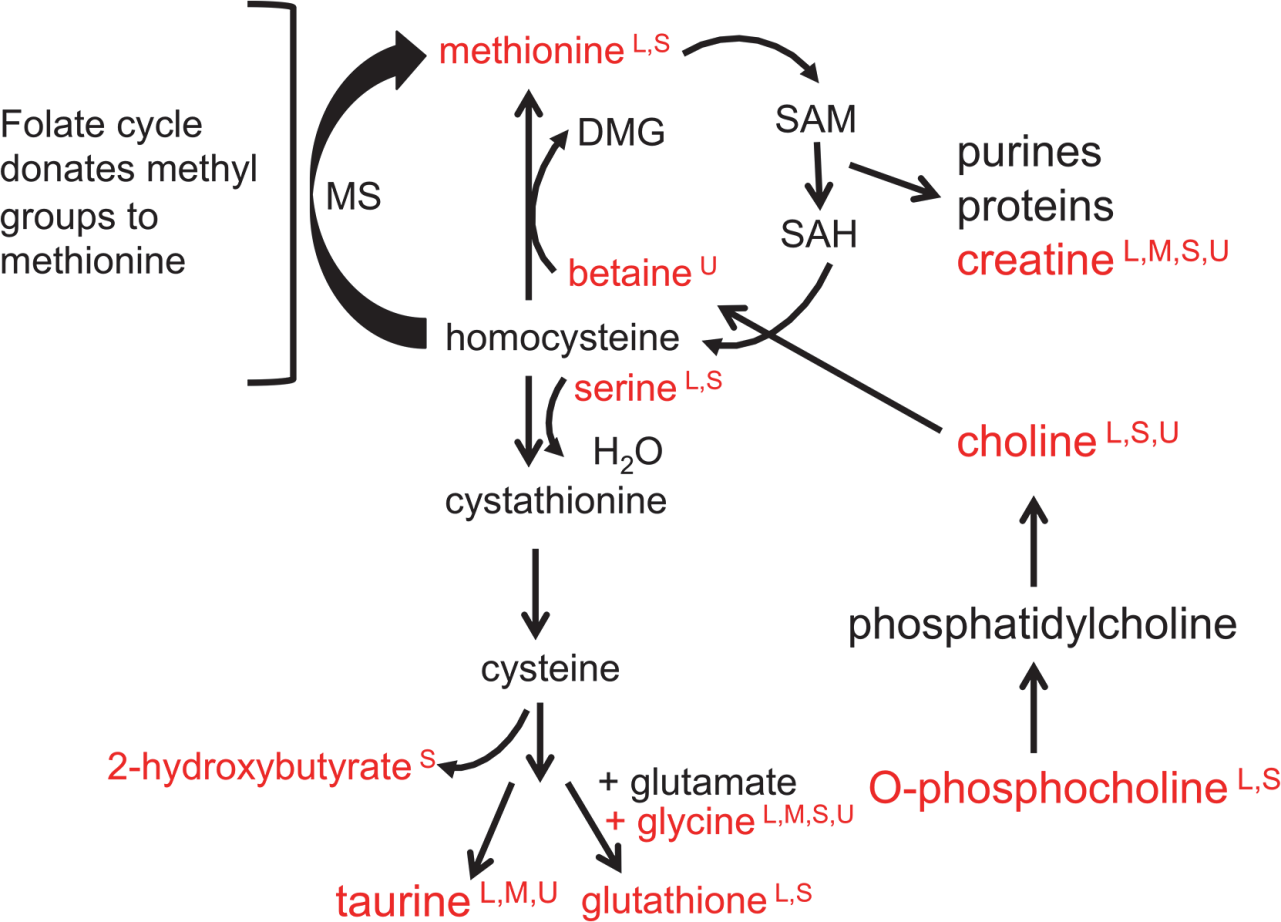
Biosynthesis involving the 1-carbon pool. Metabolomics evidence from four compartments suggests prioritization of biosynthetic activities by CPF animals at baseline. Glucose provision is associated with biosynthetic activities that involve the transfer of a methyl group. The methyl group is donated by conversion of betaine to dimethylglycine (DMG) or from the folate cycle, both actions generating S-adenosylmethionine (SAM) and S-adenosylhomocysteine (SAH). Biomolecules generated include purines, proteins, creatine, taurine, and glutathione. Metabolites in red were identified as VIP metabolites by PLS-DA analysis. Letters in superscript indicate the compartment the metabolite was observed in: liver (L), muscle (M), serum (S), or urine (U).

Higher levels of O-phosphocholine are observed in the liver of CPF animals when compared to FS animals. O-phosphocholine is a precursor of phosphatidylcholine. Phophatidylcholine can be incorporated into biological membranes or can be hydrolyzed by lipases eventually releasing choline. One biological fate of choline is oxidation to betaine (trimethylglycine) with the concurrent transfer of a methyl group to methionine. Methionine can be further processed to produce numerous biosynthetic entities such as purines and glutathione. (Fig 5) Levels of choline and betaine are higher in the urine of CPF animals when compared to FS animals. These observations support the hypothesis that phosphatidylcholine is being converted into the methyl donor betaine (via choline) for biosynthetic activities. This hypothesis would be in accordance with the use of glucose as an anabolic fuel [2]**.** Further support for increased anabolic activity comes from the ATP differential in muscle. Biosynthetic activity requires ATP. The lower level of ATP in the muscle of CPF animals, despite the greater availability of glucose, could be attributed to a higher level of biosynthetic activity in the muscle of these animals.

**Fig 6:**
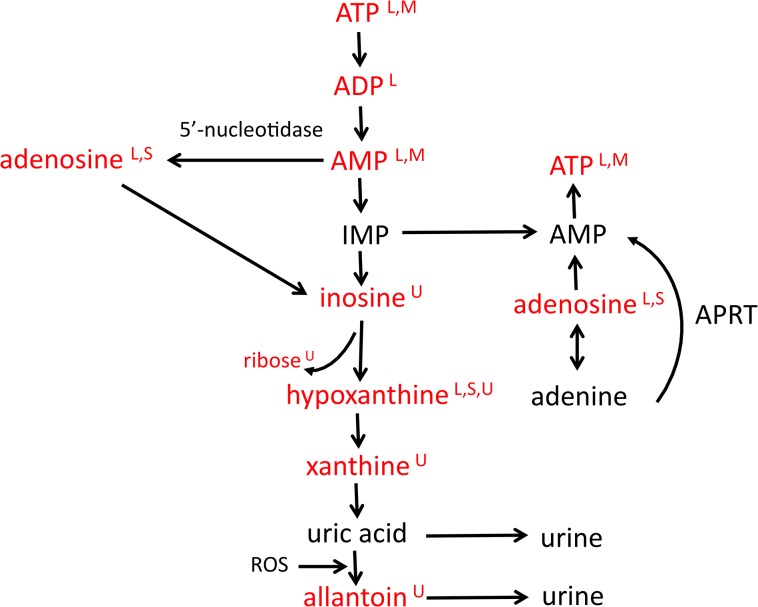
Purine degradation/salvage pathway. Metabolomics evidence from four compartments suggests alternate routes for observed differences in purine abundances. ATP is degraded in a series of reactions to uric acid or allantoin. Under non-stress conditions, this degradation process proceeds at a low level. When physiologic conditions change, intermediates in the pathway can be diverted to meet these demands. For example, during fasting, IMP can be salvaged for the production of ATP thus reducing the level of HX. In our study, HX levels are higher at baseline in liver of CPF animals. We propose that this observation is a result of increased salvage in FS animals. During ischemia 5'-nucleotidase acts on AMP to generate adenosine, a potential vasodilator. In our study, adenosine levels are ~3X higher in the liver of CPF animals when compared to FS animals suggesting a greater need for vasodilation. Metabolites in red were identified as VIP metabolites by PLS-DA analysis. Letters in superscript indicate the compartment the metabolite was observed in: liver (L), muscle (M), serum (S), or urine (U).

The level of hypoxanthine (HX) is higher in the liver of CPF animals when compared to FS animals. Liver hypoxanthine is not in the selected 10 liver metabolites, however it did exhibit a VIP score above 1.00 (VIP = 1.073) indicating its relevance. An exploration of its production provides additional support for the anabolic state associated with glucose provision. Under normal conditions (non-stress), some degradation of ATP accompanies de novo synthesis. HX is an intermediate in the degradation of ATP and gets excreted in the urine [32]. (Fig 6) The ATP degradation pathway is constructed in such a manner that intermediates can be diverted to meet altered physiologic situations. As observed in Fig 5, inosine monophosphate (IMP) can be salvaged to regenerate ATP. Enhanced purine salvage has been observed in an animal model of food deprivation [33]. At baseline levels of HX are higher in the liver of CPF animals when compared to FS animals. Although not differentiating, levels of HX are also higher by ~2 fold in the urine of CPF animals when compared to FS animals at baseline. We suggest that the difference in HX levels supports the concept of greater purine salvage in FS animals at baseline.

### 4.2. Response to Shock

**Fig 7:**
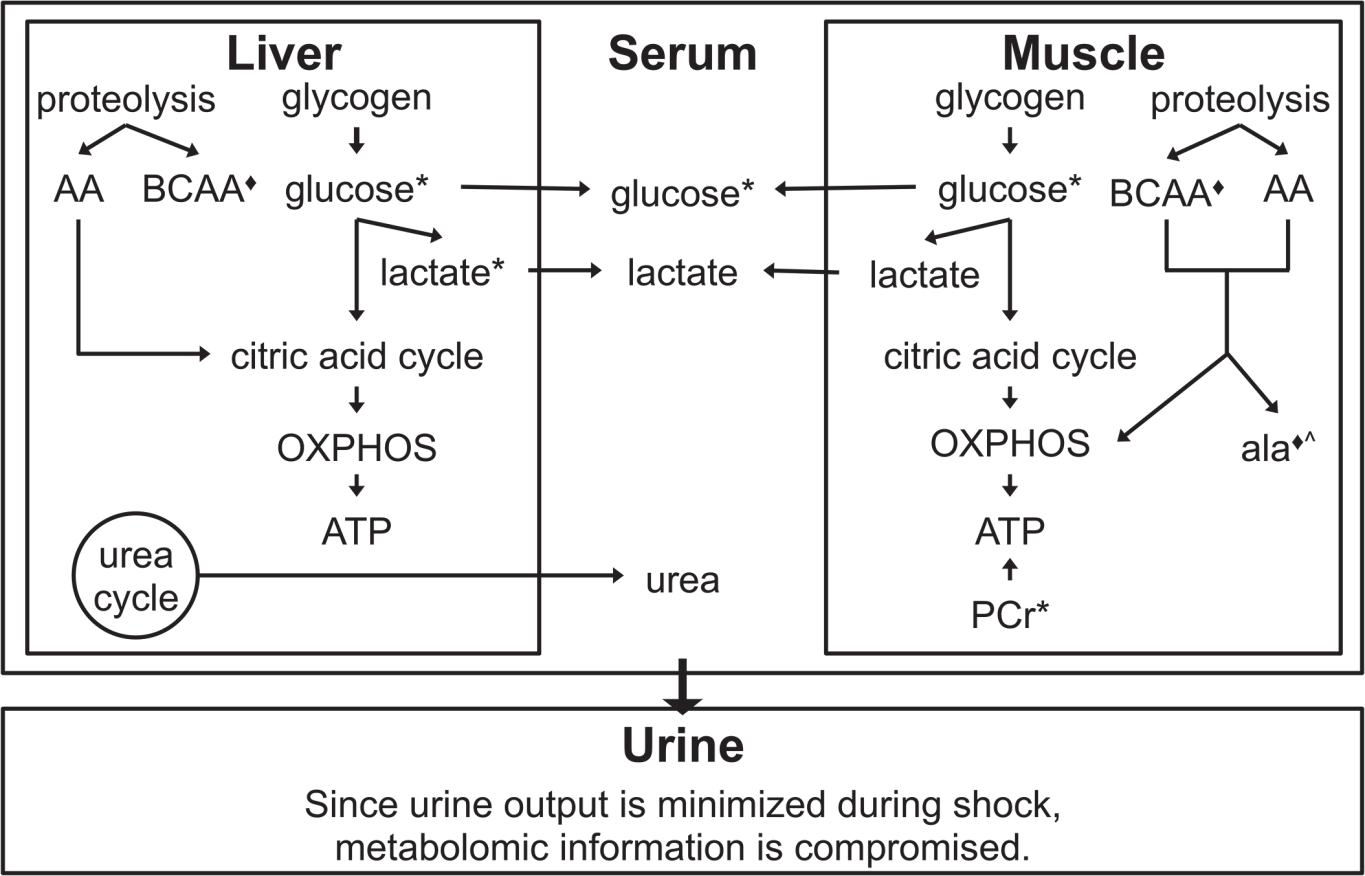
Metabolic profile associated with the response to shock. Regardless of pre-trauma dietary state, both CPF and FS animals exhibit a similar metabolic response to shock. Glucose levels increase from baseline in the liver, muscle, and serum. This is attributed to the breakdown of glycogen. The increase is greater in CPF animals presumably due to the enhanced glycogen stores in CPF animals. Both tissues also exhibit increased levels of BCAA (isoleucine, leucine, valine), suggesting more proteolysis during shock than at baseline. Greater increases in BCAA levels are observed in FS animals. Muscle creatine phosphate (PCr) levels decrease in both groups during shock compared to baseline but the decrease is greater by almost 4-fold in FS animals when compared to CPF animals. Since muscle ATP levels are not differentiating between the two groups, FS animals appeared to rely more heavily on this non-oxidative mode of ATP generation. Alanine levels (^) increase to a greater extent in the muscle of FS animals. This difference could reflect the time lag necessary to shift the metabolic machinery dedicated to glucose use in CPF animals at baseline to that necessary to process the greater load of amino acids at shock. The urine metabolome is not profiled at this time interval. During shock, animals minimize fluid loss and metabolite levels would not accurately reflect metabolic activities. * = VIP metabolite where levels observed in CPF animals > than that observed in FS animals. ◆ = VIP metabolite where levels observed in FS animals > than that observed in CPF animals.

A major finding during this time interval is the observation supporting increased reliance on internal fuel reserves in both groups (Fig 7). FS animals exhibit a drop in muscle phosphocreatine (PCr) that is nearly four times that observed in CPF animals. The change in liver HX levels differentiates CPF from FS animals and potentially arises from the greater conversion of AMP (HX precursor) to adenosine in CPF animals. Urine output is markedly reduced during the shock phase. Consequently, metabolomics information from this compartment has not been used for this time interval.

With the onset of trauma and hemorrhagic shock, glucagon is released and initiates the breakdown of glycogen stores. In CPF animals, liver, serum, and muscle levels of glucose increase from baseline. While liver and muscle glucose levels increase in FS animals as well, the level of serum glucose drops when compared to baseline. This decrease is most likely due to low glycogen levels in FS animals connected to an overnight fast [34–36]. Lactate levels increase in both liver and muscle (not differentiating) over baseline in both groups.

In addition to mobilizing glucose, increased proteolysis is observed in both groups (BCAA FS increase > BCAA CPF increase). A larger increase in the level of the gluconeogenic and/or urea precursor alanine in FS muscle parallels the BCAA pattern. Despite smaller increases of BCAA to be processed in CPF animals, levels of two BCAA catabolic products (3-methyl-2-oxovalerate originating from isoleucine and 3-hydroxyisovalerate from leucine) increase in the serum of CPF animals while showing minimal change or a decrease in FS animals. We propose that this observation reflects the time lag in CPF animals between the requirement to metabolize an increased amount of BCAA and the capacity to do so. Up to the point of shock induction, metabolic machinery in CPF animals was most likely dedicated to glucose metabolism; amino acid metabolism was supplemental.

Hypoxanthine levels decrease to a small extent in the liver of CPF animals but increase in the liver of FS. These changes result in higher HX levels in FS animals during the shock phase when compared to CPF levels (FS = 0.223, CPF = 0.197 mM/g lyophilized tissue). HX accumulates during ischemia due to the catabolism of ATP [37]. Adenosine monophosphate (AMP) is an intermediate in the catabolism of ATP. (Fig 6) During vasoconstrictive events such as hemorrhage, 5-nucleotidase hydrolyzes AMP to generate adenosine. Adenosine is a vasodilator [38]. Examination of liver adenosine levels (VIP = 0.859) reveals that the increase over baseline in CPF animals is nearly 3 times that observed in FS animals. This prioritization (diversion to adenosine production) is one potential explanation for the differences in HX concentrations.

### 4.3. Response to Resuscitation

A major finding is the diminished use of fuel (glucose and BCAA) during early resuscitation. Information evident in the urine supports greater ATP degradation in CPF animals (Fig 3).

During resuscitation the demand for fuel sources needed to mitigate the effects of hemorrhagic shock and trauma abates. With two noted exceptions (see immediately below), there is a decrease in the level of fuel sources (glucose and BCAA) in the serum, liver, and muscle of both groups. Lactate levels correspondingly decrease in the liver and serum as do downstream catabolic products of BCAAs. One exception to these trends is BCAA levels in CPF muscle. While levels of BCAAs decrease in FS muscle, they increase in the muscle of CPF animals. This observation likely arises from increased reliance on muscle proteolysis in CPF animals that accompanies the aftermath of trauma [1, 2, 3]. Although both FS and CPF animals exhibit a decrease in levels of muscle ATP at this time, the decrease in the level of FS muscle ATP is ~25 times greater than that seen in CPF animals. Further work is needed to determine the reason(s) for this.

The high demand for fuel during the shock period becomes evident in the urine during early resuscitation. Levels of glucose and a number of glucose-related metabolites (1,6-anhydro-β-D-glucose, mannose, lactose) increase in the urine (CPF increase > FS increase). (Lactate levels in the urine rise substantially during resuscitation compared to the shock period, but the levels are not differentiating.)

Levels of HX are ~2 times greater in the urine of CPF animals when compared to FS animals. It is unclear from our data why the level of HX is higher in the urine of CPF animals at this time interval. Continued degradation of HX produces uric acid that gets excreted in the urine. (Fig 6) Uric acid has the potential to induce renal dysfunction [39]. This dysfunction is correlated with an increased observation of trimethylamine-N-oxide [40]. The urine of CPF animals displays a higher level of trimethylamine-N-oxide when compared to FS animals. Greater levels of urine HX as well as trimethylamine-N-oxide suggest greater stress in CPF animals but it cannot be determined from urine data if it arises during the shock phase or during resuscitation (or both).

We recognize that a number of limitations to the above data interpretation exist. The method used for tissue extractions provides no information on the intracellular location of the metabolites. Many of the identified metabolites reside both inside and outside organelles yet the preparation method cannot distinguish between the differing locations. Secondly, metabolites identified in the biofluids (serum and urine) are reflective of the entire system. Also, an integrated picture of trauma metabolism is hindered by the fact that a metabolite observable in one compartment was not necessarily profiled in any other compartment. Finally, due to limitations of the techniques involved, we only analyzed the water-soluble metabolites present in each sample type. Glycerol may be considered a surrogate to lipids, but this was not identified as a VIP; otherwise, lipids were not considered in this study.

### Conclusions

As assessed in the liver, muscle, serum, and urine, pre-feeding (compared to an overnight fast) prior to hemorrhagic shock with injury alters the metabolic response to this trauma as well as the immediate reaction to resuscitation in a porcine model. The response to shock reflects metabolic priorities evident at baseline. FS animals raise the existing degree of proteolysis to provide additional amino acids for energy production while CPF animals rely on both glucose and, to a lesser extent, amino acids. Residual baseline activities appear to be operational during shock in CPF animals. During resuscitation, levels of metabolites associated with energy production drop, suggesting diminished demand. Additionally CPF animals exhibit a higher death rate, an observation that will be investigated in the near future. In conclusion, fed status prior to the occurrence of hemorrhagic shock with injury alters the metabolic course of this trauma and potentially affects mortality. Future studies on the etiology of hemorrhagic shock should take fed status into account.

## Supporting Information

S1 Fig PLS-DA scores plots for the baseline timepoint. PLS-DA scores plots show model discrimination between FS and CPF animals at baseline in each of the four compartments (liver, muscle, serum, urine). Models are of varying quality and statistical significance as reported in Table 1 but indicate that there is a difference in state according to feeding status.
(DOCX)

S2 Fig PLS-DA scores plots for the FR2-S45 time interval. PLS-DA scores plots show model discrimination between FS and CPF animals at baseline in each of the four compartments (liver, muscle, serum, urine). Models are of varying quality and statistical significance as reported in Table 1 but indicate that there are some differences in response to resuscitation according to feeding status.
(DOCX)

S3 Fig PLS-DA scores plots for the FR8-FR2 time interval. PLS-DA scores plots show model discrimination between FS and CPF animals at baseline in each of the four compartments (liver, muscle, serum, urine). Models are of varying quality and statistical significance as reported in S1 Table but indicate that there are some differences in response to resuscitation according to feeding status.
(DOCX)

S4 Fig PLS-DA scores plots for the FR20-FR8 time interval. PLS-DA scores plots show model discrimination between FS and CPF animals at baseline in each of the four compartments (liver, muscle, serum, urine). Models are of varying quality and statistical significance as reported in S1 Table but indicate that there are some differences in response to resuscitation according to feeding status.
(DOCX)

S1 Table Profiled Metabolites. List of metabolites profiled in each physiologic compartment.
(DOCX)

S2 Table PLS-DA model diagnostics for FR8-FR2 and FR20-FR8 time intervals. Diagnostics for PLS-DA models constructed for each physiological compartment and each timepoint or time interval discussed. Diagnostics reported are: R^2^ (indicative of the predictive utility of the model), NMC (number of misclassifications, indicative of the number of samples misclassified as FS or CPF and standard deviation), classification accuracy (indicative of the overall accuracy of sample classification as FS or CPF), and permutation p-value (indicative of model significance).
(DOCX)

S3 Table VIP (variable importance in projection) metabolites for FR8-FR2 and FR20-FR8 time intervals. Mean liver (3A) and muscle (3B) values are reported as nM/1 g lyophilyzed muscle tissue. Mean serum (3C) values are reported as mmol/L. The lab values of urea (obtained with a Gem Premier 3000 blood gas analyzer) are reported instead of NMR values since the water suppression in the CPMG pulse sequence compromises the urea signal. Mean urine (3D) values are reported as nmol/hr/kg. Bolded numbers indicate metabolites that achieved VIP scores above 1.0 or were the top 10 metabolites of those metabolites with VIP scores above 1.0.
(DOCX)
