# Supplementary material for: A Four-Compartment Metabolomics Analysis of the Liver, Muscle, Serum, and Urine Response to Polytrauma with Hemorrhagic Shock following Carbohydrate Prefeed
Source: PLoS One. 2015 Apr 14;10(4):e0124467. doi: 10.1371/journal.pone.0124467 (PMC4396978; doi:10.1371/journal.pone.0124467)
Supplement: S1 Fig — PLS-DA scores plots show model discrimination between FS and CPF animals at baseline in each of the four compartments (liver, muscle, serum, urine). Models are of varying quality and statistical significance as reported in Table 1 but indicate that there is a difference in state according to feeding status. (DOCX) [file pone.0124467.s001.docx]

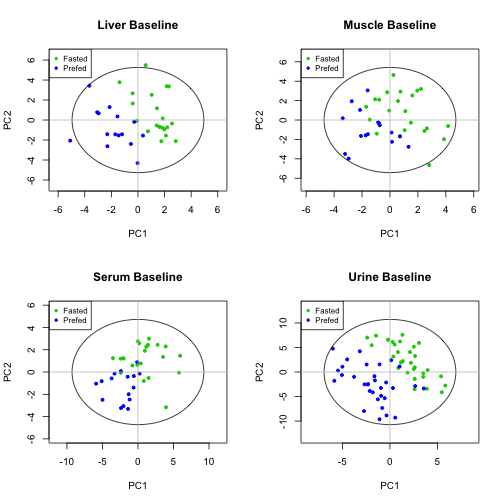


Figure S1: PLS-DA scores plots for the baseline timepoint.

PLS-DA scores plots show model discrimination between FS and CPF animals at baseline in each of the four compartments (liver, muscle, serum, urine). Models are of varying quality and statistical significance as reported in Table 1 but indicate that there is a difference in state according to feeding status.
