# Supplementary material for: A Four-Compartment Metabolomics Analysis of the Liver, Muscle, Serum, and Urine Response to Polytrauma with Hemorrhagic Shock following Carbohydrate Prefeed
Source: PLoS One. 2015 Apr 14;10(4):e0124467. doi: 10.1371/journal.pone.0124467 (PMC4396978; doi:10.1371/journal.pone.0124467)
Supplement: S4 Fig — PLS-DA scores plots show model discrimination between FS and CPF animals at baseline in each of the four compartments (liver, muscle, serum, urine). Models are of varying quality and statistical significance as reported in S1 Table but indicate that there are some differences in response to resuscitation according to feeding status. (DOCX) [file pone.0124467.s004.docx]

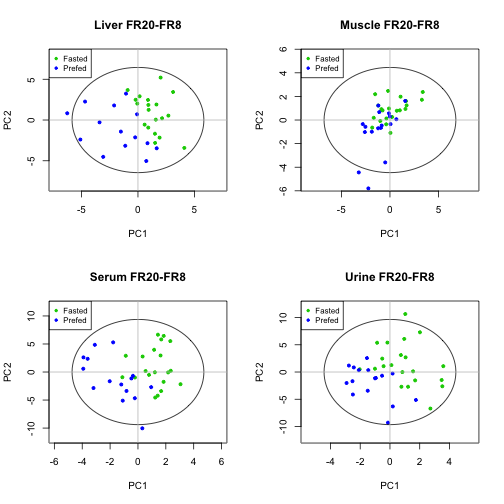


Figure S4: PLS-DA scores plots for the FR20-FR8 time interval.

PLS-DA scores plots show model discrimination between FS and CPF animals at baseline in each of the four compartments (liver, muscle, serum, urine). Models are of varying quality and statistical significance as reported in Table S1 but indicate that there are some differences in response to resuscitation according to feeding status.
