# Supplementary material for: A Four-Compartment Metabolomics Analysis of the Liver, Muscle, Serum, and Urine Response to Polytrauma with Hemorrhagic Shock following Carbohydrate Prefeed
Source: PLoS One. 2015 Apr 14;10(4):e0124467. doi: 10.1371/journal.pone.0124467 (PMC4396978; doi:10.1371/journal.pone.0124467)
Supplement: S1 Table — List of metabolites profiled in each physiologic compartment. (DOCX) [file pone.0124467.s005.docx]

Table S1: Profiled Metabolites

| Liver | | | |
| --- | --- | --- | --- |
| 3-Aminoisobutyrate | Benzoate | Histidine | Phenylalanine |
| 3-Hydroxybutyrate | Choline | Hypoxanthine | Proline |
| 3-Hydroxyisovalerate | Citrate | Isoleucine | Pyruvate |
| ADP | Creatine | Isovalerate | Serine |
| AMP | Creatinine | Lactate | sn-glycero 3-phosphocholine |
| ATP | Dimethylamine | Leucine | Succinate |
| Acetate | Formate | Lysine | Sucrose |
| Adenosine | Fumarate | Maltose | Taurine |
| Alanine | Glucose | Methionine | Tyrosine |
| Arginine | Glutamate | NAD+ | UDP Glucose |
| Asparagine | Glutamine | NADP | Valine |
| Aspartate | Glutathione | Niacinamide |  |
| Betaine | Glycine | O-Phosphocholine |  |

| Muscle | | | |
| --- | --- | --- | --- |
| 2-Oxoglutarate | Creatine | Imidazole | Pantothenate |
| 3-Hydroxybutyrate | Creatine Phosphate | Isoleucine | Pyruvate |
| 3-Hydroxyisovalerate | Formate | Lactate | Succinate |
| AMP | Fumarate | Leucine | Taurine |
| ATP | Glucose | Mannose | Valine |
| Acetate | Glutamate | Methylmalonate | myo-Inositol |
| Alanine | Glutamine | NAD+ |  |
| Carnosine | Glycine | Niacinamide |  |

| Serum | | | |
| --- | --- | --- | --- |
| 2-Aminoadipate | Arginine | Glutathione | O-Phosphocholine |
| 2-Hydroxybutyrate | Betaine | Glycerol | Phenylacetate |
| 2-Oxovalerate | Choline | Glycine | Phenylalanine |
| 3-Aminoisobutyrate | Citrate | Hippurate | Proline |
| 3-Hydroxybutyrate | Creatine | Histidine | Pyruvate |
| 3-Hydroxyisovalerate | Creatinine | Hypoxanthine | Serine |
| 3-Methyl-2-Oxovalerate | Cytidine | Isobutyrate | Succinate |
| Acetate | Dimethylamine | Isoleucine | Threonine |
| Acetoacetate | Formate | Lactate | Tyrosine |
| Adenosine | Glucose | Leucine | Uridine |
| Adipate | Glutamate | Mannose | Valine |
| Alanine | Glutamine | Methionine |  |

| Urine | | | |
| --- | --- | --- | --- |
| 1,6 Anhydro β-D glucose | Creatine | Lactate | Taurine |
| 1-Methylnicotinamide | Creatinine | Lactose | Thymine |
| 2-Ethylacrylate | Cytosine | Malonate | Tiglylglycine |
| 2-Methylglutarate | Dimethylamine | Mannitol | Trigonelline |
| 2-Oxoglutarate | Ethanolamine | Mannose | Trimethylamine |
| 3-Hydroxyisovalerate | Formate | Methylguanidine | Trimethylamine N-oxide |
| 3-Methylxanthine | Fumarate | N,N-Dimethylglycine | Tryptophan |
| 4-Aminohippurate | Glucose | N-Methylhydantoin | Tyramine |
| Acetate | Glycine | N-Phenylacetylglycine | Tyrosine |
| Acetoacetate | Hippurate | Niacinamide | Urocanate |
| Acetone | Histamine | Phenylacetylglycine | Xanthine |
| Alanine | Histidine | Phenylalanine | cis-Aconitate |
| Allantoin | Homogentisate | Pyruvate | pi-Methylhistidine |
| Betaine | Hypoxanthine | Quinolinate |  |
| Choline | Inosine | Ribose |  |
| Citrate | Isovalerate | Succinate |  |

Table S1: List of metabolites profiled in each physiologic compartment.
