# Supplementary material for: A Four-Compartment Metabolomics Analysis of the Liver, Muscle, Serum, and Urine Response to Polytrauma with Hemorrhagic Shock following Carbohydrate Prefeed
Source: PLoS One. 2015 Apr 14;10(4):e0124467. doi: 10.1371/journal.pone.0124467 (PMC4396978; doi:10.1371/journal.pone.0124467)
Supplement: S2 Table — Diagnostics for PLS-DA models constructed for each physiological compartment and each timepoint or time interval discussed. Diagnostics reported are: R2 (indicative of the predictive utility of the model), NMC (number of misclassifications, indicative of the number of samples misclassified as FS or CPF and standard deviation), classification accuracy (indicative of the overall accuracy of sample classification as FS or CPF), and permutation p-value (indicative of model significance). (DOCX) [file pone.0124467.s006.docx]

Table S2: PLS-DA model diagnostics for FR8-FR2 and FR20-FR8 time intervals.

| Model | Compartment | R^2^ | NMC ± std. dev. | Classification Accuracy | Permutation p-value |
| --- | --- | --- | --- | --- | --- |
| FR8-FR20 | Liver | 0.630 | 3.312±1.21 | 93.94% | 0.149 |
|  | Muscle | 0.234 | 4.212±1.30 | 86.84% | 0.860 |
|  | Serum | 0.585 | 3.270±1.23 | 97.14% | 0.029 |
|  | Urine | 0.493 | 3.324±1.26 | 100% | 0.005 |
| FR20-FR8 | Liver | 0.591 | 3.505±1.14 | 96.97% | 0.036 |
|  | Muscle | 0.300 | 3.918±1.29 | 76.92% | 1.00 |
|  | Serum | 0.632 | 2.614±1.22 | 100% | 0.005 |
|  | Urine | 0.558 | 4.003±1.27 | 100% | 0.007 |

Diagnostics for PLS-DA models constructed for each physiological compartment and each timepoint or time interval discussed. Diagnostics reported are: R^2^ (indicative of the predictive utility of the model), NMC (number of misclassifications, indicative of the number of samples misclassified as FS or CPF and standard deviation), classification accuracy (indicative of the overall accuracy of sample classification as FS or CPF), and permutation p-value (indicative of model significance).
