# Supplementary material for: A Four-Compartment Metabolomics Analysis of the Liver, Muscle, Serum, and Urine Response to Polytrauma with Hemorrhagic Shock following Carbohydrate Prefeed
Source: PLoS One. 2015 Apr 14;10(4):e0124467. doi: 10.1371/journal.pone.0124467 (PMC4396978; doi:10.1371/journal.pone.0124467)
Supplement: S3 Table — Mean liver (3A) and muscle (3B) values are reported as nM/1 g lyophilyzed muscle tissue. Mean serum (3C) values are reported as mmol/L. The lab values of urea (obtained with a Gem Premier 3000 blood gas analyzer) are reported instead of NMR values since the water suppression in the CPMG pulse sequence compromises the urea signal. Mean urine (3D) values are reported as nmol/hr/kg. Bolded numbers indicate metabolites that achieved VIP scores above 1.0 or were the top 10 metabolites of those metabolites with VIP scores above 1.0. (DOCX) [file pone.0124467.s007.docx]

Table S3: VIP (variable importance in projection) metabolites for FR8-FR2 and FR20-FR8 time intervals.

| **Table S3A** | **FR8-FR2** | | **FR20-FR8** | |
| --- | --- | --- | --- | --- |
| **Liver Metabolite** | **CPF** | **FS** | **CPF** | **FS** |
| benzoate | **0.10** | **-0.03** | **-0.09** | **0.02** |
| UDP glucose | **-0.13** | **0.08** | 0.09 | 0.08 |
| ATP | **0.21** | **0.06** | -0.14 | -0.03 |
| S-adenosylhomocysteine | **0.08** | **-0.05** | -0.08 | -0.03 |
| ADP | **0.10** | **0.01** | -0.05 | 0.08 |
| arginine | **0.23** | **-0.06** | -0.36 | -0.07 |
| isovalerate | **0.004** | **-0.004** | 0.005 | 0.003 |
| glutathione | **0.08** | **0.48** | -0.20 | 0.24 |
| hypoxathine | **0.008** | **-0.06** | -0.16 | -0.007 |
| creatine | **0.41** | **1.3** | 0.72 | 0.07 |
| Sn-glycero-3-phosphocholine | -1.3 | -1.4 | **0.09** | **1.69** |
| succinate | 0.00008 | -0.02 | **-0.08** | **-0.003** |
| alanine | -0.27 | -0.62 | **-1.11** | **-0.17** |
| O-phosphocholine | -0.04 | -0.03 | **-0.74** | **-0.02** |
| choline | 0.19 | -0.03 | **-0.35** | **-0.02** |
| dimethylamine | 0.03 | 0.009 | **-0.04** | **-0.003** |
| citrate | 0.02 | 0.04 | **-0.04** | **0.08** |
| isoleucine | -0.00009 | -0.02 | **0.02** | **-0.02** |
| valine | -0.01 | -0.03 | **0.009** | **-0.04** |
|  |  | |  | |
| **Table S3B** | **FR8-FR2** | | **FR20-FR8** | |
| **Muscle Metabolite** | **CPF** | **FS** | **CPF** | **FS** |
| ATP | **-1.2** | **0.43** | **0.14** | **-1.3** |
| glycine | **-0.38** | **0.02** | -0.01 | -0.15 |
| taurine | **-1.6** | **1.1** | **-1.4** | **-4.2** |
| isoleucine | **-0.03** | **0.02** | **0.03** | **-0.04** |
| creatine | **-2.5** | **2.3** | **-1.3** | **-5.7** |
| 3-hydroxyisovalerate | **-0.007** | **0.01** | **-0.007** | **-0.01** |
| 2-oxoglutarate | **-0.08** | **0.05** | -0.18 | -0.24 |
| creatine phosphate | **-4.1** | **-2.0** | -0.12 | -2.1 |
| carnosine | **-1.3** | **0.41** | -0.18 | -1.5 |
| myo-inositol | **-0.11** | **0.13** | **-0.14** | **-0.47** |
| valine | -0.03 | 0.3 | **0.003** | **-0.09** |
| formate | 0.05 | 0.07 | **0.09** | **-0.03** |
| 3-hydroxybutyrate | -0.02 | -0.03 | **0.02** | **-0.001** |
| glutamine | -0.21 | 0.03 | **-1.1** | **-2.0** |
|  |  | |  | |
| **Table S2C** | **FR8-FR2** | | **FR20-FR8** | |
| **Serum Metabolite** | **CPF** | **FS** | **CPF** | **FS** |
| formate | **0.45** | **-0.09** |  |  |
| isobutyrate | **-0.01** | **-0.03** |  |  |
| creatinine | **0.02** | **-0.03** | **-0.08** | **-0.05** |
| creatine | **0.47** | **0.02** | -0.61 | -0.62 |
| arginine | **-0.06** | **-0.37** | -0.42 | -0.47 |
| glutamate | **-0.08** | **-0.38** | -0.42 | -0.36 |
| tyrosine | **-0.03** | **-0.09** | -0.13 | -0.14 |
| pyruvate | **-0.19** | **-0.44** | **-0.23** | **-0.14** |
| valine | **-0.11** | **-0.3** | -0.30 | -0.37 |
| citrate | **-0.03** | **-0.15** | **-0.08** | **-0.04** |
| 2-oxovalerate | 0.02 | 0.03 | **0.19** | **0.65** |
| adipate | 0.03 | 0.01 | **1.02** | **0.28** |
| adenosine | -0.001 | 0.004 | **0.006** | **-0.005** |
| glucose | -0.54 | -4.6 | **-15.3** | **-9.3** |
| alanine | -0.51 | -1.2 | **-1.04** | **-0.71** |
| threonine | -0.02 | -0.07 | **-0.21** | **-0.34** |
| isoleucine | -0.09 | -0.15 | **-0.08** | **-0.13** |
|  |  |  |  |  |
| **Table S3D** | **FR8-FR2** | | **FR20-FR8** | |
| **Urine Metabolite** | **CPF** | **FS** | **CPF** | **FS** |
| creatine | **1124** | **4083** | 2431 | 3460 |
| glucose | **-68173** | **-7023** | 3794 | 1943 |
| mannose | **-318** | **-5.1** | -48 | -49 |
| glycine | **164** | **1036** | **1438** | **780** |
| hypoxanthine | **-779** | **-138** | -136 | -187 |
| mannitol | **-175** | **370** | 168 | -25 |
| urocanate | **-6.0** | **39** | **66** | **19** |
| quinolinate | **80** | **173** | **-45** | **-105** |
| trigonelline | **-3.3** | **16** | -5.3 | -4.4 |
| dimethylamine | **113** | **330** | 129 | 46 |
| 1,6 anhydro-βD glucose | -130 | 33 | **-1472** | **7.9** |
| 1-methylnicotinamide | -58 | -65 | **-5.0** | **-17** |
| acetate | -67 | -175 | **-18** | **-47** |
| taurine | 725 | 1510 | **3584** | **1917** |
| acetoacetate | 149 | 298 | **-34** | **351** |
| 4-aminohippurate | 30 | 85 | **30** | **-28** |
| 3-methylxanthine | 67 | 18 | **-56** | **42** |

Table S3: VIP (variable importance in projection) metabolites for FR8-FR2 and FR20-FR8 time intervals.

Mean liver (3A) and muscle (3B) values are reported as nM/1 g lyophilyzed muscle tissue. Mean serum (3C) values are reported as mmol/L. The lab values of urea (obtained with a Gem Premier 3000 blood gas analyzer) are reported instead of NMR values since the water suppression in the CPMG pulse sequence compromises the urea signal. Mean urine (3D) values are reported as nmol/hr/kg. Bolded numbers indicate metabolites that achieved VIP scores above 1.0 or were the top 10 metabolites of those metabolites with VIP scores above 1.0.
